# Thoracic 9 Spinal Transection-Induced Model of Muscle Spasticity in the Rat: A Systematic Electrophysiological and Histopathological Characterization

**DOI:** 10.1371/journal.pone.0144642

**Published:** 2015-12-29

**Authors:** Jose A. Corleto, Mariana Bravo-Hernández, Kota Kamizato, Osamu Kakinohana, Camila Santucci, Michael R. Navarro, Oleksandr Platoshyn, Dasa Cizkova, Nadezda Lukacova, Julian Taylor, Martin Marsala

**Affiliations:** 1 Neuroregeneration Laboratory, Department of Anesthesiology, University of California - San Diego, La Jolla, California, United States of America; 2 Biomedical Sciences Graduate Program University of California San Diego, La Jolla, California, United States of America; 3 Department of Pharmacobiology, Centro de Investigacion y de Estudios Avanzados Cinvestav) Sede Sur, Mexico D.F., Mexico; 4 Hospital Nacional de Paraplejicos, SESCAM, Toledo, Spain; 5 Institute of Neurobiology, Slovak Academy of Sciences, Soltesovej 6, Kosice, Slovakia; University of Toronto, CANADA

## Abstract

The development of spinal hyper-reflexia as part of the spasticity syndrome represents one of the major complications associated with chronic spinal traumatic injury (SCI). The primary mechanism leading to progressive appearance of muscle spasticity is multimodal and may include loss of descending inhibitory tone, alteration of segmental interneuron-mediated inhibition and/or increased reflex activity to sensory input. Here, we characterized a chronic thoracic (Th 9) complete transection model of muscle spasticity in Sprague-Dawley (SD) rats. Isoflurane-anesthetized rats received a Th9 laminectomy and the spinal cord was transected using a scalpel blade. After the transection the presence of muscle spasticity quantified as stretch and cutaneous hyper-reflexia was identified and quantified as time-dependent changes in: **i)** ankle-rotation-evoked peripheral muscle resistance (PMR) and corresponding electromyography (EMG) activity, **ii)** Hoffmann reflex, and **iii)** EMG responses in gastrocnemius muscle after paw tactile stimulation for up to 8 months after injury. To validate the clinical relevance of this model, the treatment potency after systemic treatment with the clinically established anti-spastic agents baclofen (GABA_B_ receptor agonist), tizanidine (α_2_-adrenergic agonist) and NGX424 (AMPA receptor antagonist) was also tested. During the first 3 months post spinal transection, a progressive increase in ankle rotation-evoked muscle resistance, Hoffmann reflex amplitude and increased EMG responses to peripherally applied tactile stimuli were consistently measured. These changes, indicative of the spasticity syndrome, then remained relatively stable for up to 8 months post injury. Systemic treatment with baclofen, tizanidine and NGX424 led to a significant but transient suppression of spinal hyper-reflexia. These data demonstrate that a chronic Th9 spinal transection model in adult SD rat represents a reliable experimental platform to be used in studying the pathophysiology of chronic spinal injury-induced spasticity. In addition a consistent anti-spastic effect measured after treatment with clinically effective anti-spastic agents indicate that this model can effectively be used in screening new anti-spasticity compounds or procedures aimed at modulating chronic spinal trauma-associated muscle spasticity.

## Introduction

The progressive development of muscle spasticity represents a serious complication associated with chronic traumatic spinal cord injury. By definition the spasticity is characterized by the presence of muscle-stretch-velocity-dependent increase in muscle resistance. In addition to a progressive appearance of muscle spasticity several other qualitatively distinct neurophysiological or functionally-defined deficits including exaggerated tendon reflex or muscle clonus are frequently seen in patients with chronic spinal trauma. In general all these pathological states are believed to be the result of spinal hyper-reflexia when peripherally applied stimuli (such as muscle stretch or tactile/thermal stimuli) lead to an exacerbated EMG response in corresponding segmental dermatomes [1]. Reflex activity evoked by electrical plantar stimulation in patients has been shown to be higher in patients with spinal cord injury spasticity syndrome [2]. Systematic clinical data show that 29–50% patients with chronic spinal trauma show the development of variable degree of spasticity [3]. Importantly, the presence of muscle spasticity often represents a major limiting factor in achieving a clinically relevant motor recovery in patients with incomplete spinal cord injuries even if continuing and aggressive post-injury physical rehabilitation regimen is maintained [4, 5]. As such the development of new anti-spastic therapies, in conjunction with physical rehabilitation, are critical in leading to a more effective functional recovery in incomplete spinal trauma patients.

The mechanism leading to development of muscle spasticity after spinal injury is multifactorial. It is believed that one of the primary mechanism is the loss of descending tonic inhibition (resulting from the loss of descending tracts integrity) and resulting decrease in GABA/glycine-ergic pre-synaptic inhibition in segments below the level of SCI [6–8]. Second, the local spinal injury may lead to a significant activation of glial elements (astrocytes, microglia), corresponding release of proinflammatory cytokines and resulting activation of local excitatory neurons through a secondary excitatory amino acid (glutamate) release [9–20]. It is important to note that the degree of glial cell activation may vary significantly depending of the model or mechanism of spinal injury/degeneration employed (spinal trauma, ischemia, amyotrophic lateral sclerosis, multiple sclerosis). Therefore a relative contribution of these proinflammatory processes in the evolution of spasticity state can vary and needs to be defined for each specific model.

Over past decades several spinal trauma models of spasticity, i.e. the most relevant models to our current study have been reported. **First,** a complete S2 transection model of tail muscle spasticity in adult SD rat was developed [21]. In this model a time-dependent appearance of muscle stretch-evoked muscle spasticity (as measured by changes in tail resistance and EMG) was demonstrated. Importantly, similarly as seen in clinical patients with chronic muscle spasticity the presence of extremely developed hyper-reflexia to a light touch or peripherally applied thermal stimuli was also seen. **Second,** selective dorsolateral lesion of the lower thoracic or upper lumbar spinal cord has been shown to develop an increase in stretch-evoked reflex activity [22] with a reduction in threshold and increase in reflex gain in the awake trained cat [23]. **Third,** a complete Th10 spinal transection model of hindlimb spasticity in adult rat was recently developed. To identify the presence of spinal hyper-reflexia, changes in ankle torque and the corresponding increase in gastrocnemius muscle EMG activity was used. A significant presence of spasticity was seen at 6 weeks after spinal transection. In addition the loss of rate-dependent depression of the Hoffmann reflex was measured [8, 24, 25]. While not so pronounced the presence of muscle spasticity was also reported after spinal Th10 contusion injury in rat. Using a high velocity of ankle rotation (400/s) an increased ankle torque and EMG activity was measured at 7–14 days after trauma [26–28].

We have recently developed a computer-controlled ankle rotational device which permits an objective measurement of ankle resistance and corresponding changes in EMG activity measured in gastrocnemius muscle in fully awake restrained rats [29]. Using this system coupled with rat models of spinal ischemic injury, L3 compression injury or spinal air-embolism-induced injury we have demonstrated the presence of velocity-dependent increase in muscle resistance which correlates with an increased EMG activity in gastrocnemius muscle [28–30]. In subsequent studies using the spinal ischemic model and spinal air embolism model of muscle spasticity we have demonstrated: **i)** a potent anti-spastic effect after spinal or systemic treatment with baclofen [31], tizanidine [32], NGX424 [13], **ii)** the effect of a combined anti-spastic therapy after spinal upregulation of GAD65 gene and systemic treatment with tiagabine [33], and, **iii)** development of baclofen tolerance in rats infused intrathecally with baclofen using a miniosmotic pump [34] and effective suppression of spasticity in baclofen-tolerant animals after systemic treatment with AMPA receptor antagonist NGX424 [35].

To expand our previous studies which employed the rat spinal-ischemic, spinal air embolism and compression injury models, we now characterize the time course of muscle spasticity induced by a complete spinal cord (Th9) transection. The presence of spinal hyper-reflexia was identified by: **i)** changes in ankle resistance and gastrocnemius EMG activity during progressively increased velocities of ankle rotation, **ii)** changes in gastrocnemius muscle EMG response after peripheral tactile or thermal stimuli, and **iii)** changes in the Hoffmann reflex. In addition, to validate the clinical relevance of this spasticity model we characterized the potency of systematically delivered clinically effective anti-spastic agents including baclofen (GABA_B_ agonist), tizanidine (α_2_ adrenergic agonist) and NGX424 (a novel AMPA receptor antagonist) in animals at chronic stages of spinal transection-induced spasticity. Finally, spinal immunofluorescence qualitative and quantitative analysis was performed and the expression of several neuronal/non-neuronal markers and neurotransmitter(s) transporter systems were analyzed including NeuN, GAD65/67, GFAP, Iba1, VGluT1/VGluT2 (vesicular glutamate transporters) and GlyT2 (Glycine transporter 2).

## Material and Methods

### T8 laminectomy and T9 spinal segment transection

All procedures were approved by the Institutional Animal Care and Use Committees by the University of California, San Diego. Adult Sprague-Dawley rats (Male and female, 250–350 grams) were used. Rats were anesthetized with 5% isoflurane and maintained at 2–3% of isoflurane during surgery depending on breathing rate and paw pinch response. The back of rat was then shaved and cleaned with 2% chlorohexadine. After skin incision the paravertebral muscle surrounding the Th8 vertebra was removed and animals mounted into spinal immobilization frame (Stoelting) using Cunningham’s spinal clamps. To expose the Th9 spinal segment a dorsal laminectomy of Th8 vertebra was performed using dental drill. A bent 30 gauge needle was then used to cut the spinal dura overlaying the Th9 segment. The spinal cord was then ‘hooked’ epidurally using a spinal hook, and a #11 surgical blade was used to perform a complete transdural spinal transection; the completeness of spinal cord transection was confirmed according to the ability to retract the spinal hook across the transected spinal cord tissue. After spinal cord transection the incision site was cleaned with Pencillin/Streptomycin solution (10,000 Units/mL: 10,000 micrograms/mL), paravertebral muscles sutured using 4–0 absorbable suture and the skin incision closed using staples. Animals were then allowed to recover. All animals were treated for 10–12 days with: Cefazolin (10 mg/kg/day, SC) and post-surgical pain control was performed with buprenorphine (0.05 mg/kg SC; 2–3 days).

### Drugs

Baclofen ((±)-β-(Aminomethyl)-4-chlorobenzenepropanoic acid), Tizanidine (5-Chloro-N-(4,5-dihydro-1H-imidazol-2-yl)-2,1,3-benzothiadiazol-4-amine hydrochloride) were purchased from Sigma- Aldrich (St. Louis, MO). NGX424 (Tezampanel, (3S,4aR,6R,8aR)-6-[2-(1H-tetrazol-5-yl)ethyl]decahydroisoquinoline-3-carboxylic acid) was a generous gift provided by Torrey Pines Therapeutics, Inc. All drugs were diluted in saline prior to administration. Ketaset^®^ 1000mg/10 mL (Ketamine, (RS)-2-(2-clorofenyl)-2-(metilamino) ciclohexan-1-ona) was purchased on Fort Dodge (Iowa, USA). Cefazolin 1g ampoules ((6R,7R)-3-{[(5-methyl-1,3,4-thiadiazol-2-yl)thio]methyl}-8-oxo-7-[(1H-tetrazol-1-ylacetyl)amino]-5-thia-1-azabicyclo[4.2.0]oct-2-ene-2-carboxylic acid) bought from Sandoz Inc (NJ, USA). Pencillin/Streptomycin was purchased from Gibco, Life technologies (NY, USA). Sulfametoxazol and Trimethoprim 200mg/40mg per 5 mL was purchased from Hi-Tech Pharmacal (NY, USA).

### Measurement of muscle spasticity using computer-controlled ankle-rotational system

**Fig 1:**
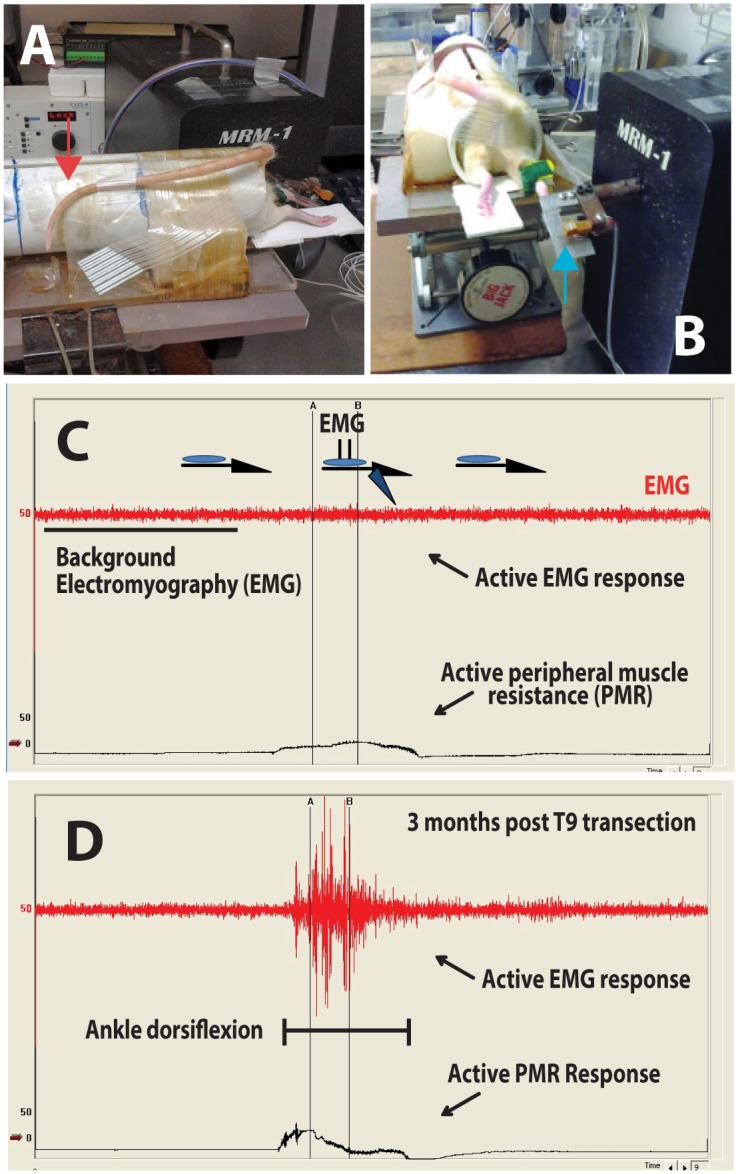
Experimental setup to permit the measurement of computer-controlled ankle dorsiflexion-evoked muscle resistance and corresponding changes in gastrocnemius muscle EMG activity in fully awake restrained animals. **(A, B)**—Fully awake rats are placed into PVC tube (internal diameter: 6 cm; red arrow) and the right (or left) paw is attached to a bridging pressure transducer (blue arrow). During computer-controlled ankle rotation the changes in ankle resistance (active peripheral muscle resistance = PMR) is recorded. At the same time the EMG (active EMG response) is recorded from gastrocnemius muscle by using transcutaneously placed needle recording electrodes. **(C, D)**- An example of EMG and PMR recording before, during and after computer-controlled ankle dorsiflexion (0→80°; 400°/sec) in naïve-non-injured rat or in an animal at 3 months post-Th9 transection (D). Note a near complete EMG and PMR non-responsiveness during ankle dorsiflexion in naive-non-injured animal (C) but a clear burst like-EMG activity and corresponding increase in PMR in chronically transected animal (D).

To identify the presence of muscle spasticity in rats after spinal transection previously developed computer-controlled ankle-rotational device was used [29] Briefly, fully awake animals were restrained using a PVC pipe (6 cm x 30 cm; Fig 1A-red arrow) and their right paw attached to a paw attachment metal plate (Fig 1B-blue arrow). Paw attachment plate is bridged to a stepping motor using digital pressure transducer permitting the measurement of ankle resistance during computer-controlled ankle rotation. To measure spasticity the ankle was rotated to either 40° or 80° from baseline position at rotational velocities of 40 degrees per seconds (40° s^−1^, DPS), 200 DPS or 400 DPS. Before, during and after ankle rotation the ankle resistance (peripheral muscle resistance = PMR) and gastrocnemius muscle EMG (active EMG response) was recorded.

### Hoffmann reflex (H-reflex) and Rate-dependent depression recordings

H-reflex was recorded as previously described [31]. Briefly, under ketamine anesthesia (100 mg/kg/h, i.m.), the right hind limb of the animal was secured, and a pair of stimulating needle electrodes was transcutaneously inserted into the surroundings of the tibial nerve. For recording, a pair of silver needle electrodes was placed into the interosseous muscles between the fourth and the fifth, or the first and the second metatarsal right foot muscles. The tibial nerve was stimulated using square pulses with increasing stimulus intensity (0.1–10 mA in 0.5 mA increments, 0.1 Hz, 0.2 ms; WPI; Isostim A320), and responses were recorded with an A/C-coupled differential amplifier (Model DB4; DPI, Sarasota, FL, USA). The threshold for both the M and H waves was determined and Hmax/Mmax ratio calculated. To measure rate-dependent depression the tibial nerve was stimulated with a series of 20 stimulus impulses at 0.1, 0.5, 1.0, 5.0, and 10 Hz and the changes in H wave compared at different stimulation frequencies.

### Von Frey Tactile Response Measurements

Both transected (3 months post-transection) and age-matched control rats were restrained in a modified PVC tube with the exposed hind paws taped to a wooden board (S2 Fig). The right hind leg had all the hair removed and surface EMG recording electrodes (Ambu; Neuroline 700) were placed along the gastrocnemius muscle. The plantar surface of the paw was exposed and von Frey filaments ranging from 1 gram to 15 grams of pressure were used to apply a pressure at the base of third digit in fully awake animals. Other studies have previously reported that paw pressure of up to 15g have a minimal or no response on paw retraction in control rats [36]. Two different testing paradigms were used. **First**, the EMG response was measured after applying 5 consecutive stimuli of the same paw pressure when stimuli were separated by 10–15 sec intervals. The paw pressure was then increased and the test of 5 consecutive stimuli repeated until a supramaximal response was seen. Second, the EMG response was measured after applying 5 consecutive stimuli of the same paw pressure when stimuli were separated by exactly 5 sec intervals. The paw pressure was then increased and the test of 5 consecutive stimuli delivered every 5 sec repeated until a supramaximal response was seen. The goal of this higher frequency stimulation was to determine if there is any potentiation or suppression of evoked EMG response. For quantitative analysis an averaged, integrated EMG signal (5 sec) recorded after 5 stimuli delivered at the same pressure intensity was used.

### Assessment of the potency of anti-spastic agents in rats with developed spasticity

Spastic rats at 3 months post-transection were implanted with intraperitoneal PE-10 catheter for drug delivery. Three days after catheter implant the baseline spasticity response was measured using ankle-rotational system. Animals were the injected with either Baclofen (Sigma, 10 mg/kg), Tizanidine (Sigma, 1 mg/kg), or NGX424 (Tezampanel, TorreyPines therapeutics, Inc., 1 mg/kg) delivered as a single bolus injection over 60 sec interval. After treatment the anti-spasticity effect was measured in 5 minute intervals for two hours. In a separate recording sessions the effect of identical treatments on the H-reflex and tactile stimulus-evoked hyper-reflexia was tested at 1 hr post-injection.

### Perfusion, fixation and immunofluorescence staining of spinal cord sections

Rats were deeply anesthetized with pentobarbital and transcardially perfused with 200 ml of heparinized saline followed by 250 ml of 4% paraformaldehyde in PBS. The spinal cords were dissected and post-fixed in 4% formaldehyde in PBS overnight at 4°C and then cryoprotected in 30% sucrose PBS until transverse or longitudinal sections (30-μm-thick) were cut on a cryostat and stored in PBS. Sections were immunostained overnight at 4°C with the following primary antibodies made in PBS with 0.2% Triton X-100: Goat anti-Choline acetyltransferase (ChAT; 1:100; Chemicon), guinea pig anti-vesicular glutamate 1 (VGluT1, 1:1500), chicken anti-glial fibrillary acidic protein (GFAP-Cy3; 1:1000, Sigma-Aldrich), mouse anti-neuronal nuclei antigen (NeuN, 1:1000, Chemicon), rabbit anti-GAD65/67 (1:1000, EMD Millipore), mouse anti-synaptophysin (1:200, Vector), rabbit anti-Iba1 (1:1000, Wako). After incubation with primary antibodies, sections were washed three times in PBS and incubated with fluorescent-conjugated secondary donkey anti-guinea pig, donkey anti-rabbit, donkey anti-mouse or donkey anti-goat antibodies respectively (Alexa Fluor 488, 546 or 647, 1:1000, Invitrogen) and DAPI for general nuclear staining. Sections were then mounted on slides, dried at room temperature and covered with a Prolong anti-fade kit (Invitrogen). Fluorescence images were captured using a Zeiss Imager M2 microscope with Stereo investigator software and confocal images were taken using an Olympus FV1000 microscope.

### Quantification of NeuN, GlyT2, GAD65/67, GFAP, Iba1 and VGluT1-stained sections

For quantitative analysis a total of 4 transected animals at 3 months post-transection and 4 age-matched controls were used. L2-L6 spinal segments were serially cut (frozen transverse 30`m thick sections) and 3 sections per each segmental level (i.e. total of 15 sections per animal) collected. To validate the segment level of collected sections the laminar morphology of each section was compared to the spinal segmental laminar map and cell morphology as published by Paxinos [37].

The images were captured on a monochromatic Zeiss AxioCam at the same exposure settings and fluorescent lamp intensity. Mosaic images were captured from one half of the spinal cord at the desired wavelength/spectrum of light. For NeuN quantification, Photoshop was used to crop a section of one half of the lumbar spinal cord (at 10X) to the left or right of the central canal measuring 937 by 432 pixels (Width x Height) corresponding to lamina VII. For VGluT1 staining the entire ventral horn of the spinal cord was cropped 90 degrees from the central canal. Image J was used to quantify NeuN positive cells and VGluT1 puncta by first converting image to black and white binary and the watershed function was employed to separate neurons that were proximal to one another from being considered one large fluorescent signal. The signal of NeuN staining was then quantified by setting the analyze particles sub menu to Size (pixel^2) 50-Infinity at a circularity of 0.00–1.00, while the VGluT1 staining was performed with a Size (pixel^2) of 30-Infinity at the same circularity. The results and summary were then selected for display on this menu and the count then being used to represent each respective region of the lumbar spinal segment. GlyT2, GAD65/67, GFAP, and Iba1 signal density was quantified by capturing images at 10X of the entire ventral horn 90 degrees to the central canal. Image J software was then used to calculate the average mean gray value in the whole ventral horn. The average signal intensity from each respective marker/staining was taken from the naïve, non-injured rats and was considered as 100% value. Mean gray values measured in T9 TSCT animals were then compared to naïve animals and expressed as % naïve animal signal intensity.

### Statistical Analysis

Statistical analysis of the integrated-averaged EMG and PMR responses were analyzed by one way ANOVA with Bonferroni correction. The H reflex and tactile stimulus-evoked EMG data were analyzed using an unpaired student’s t test. The RDD data and the effect of pharmacology treatment on PMR, EMG and tactile stimulus-evoked EMG response were analyzed by two way ANOVA with Bonferroni post hoc correction.

## Results

### Time-dependent appearance of muscle spasticity after spinal transection

**Fig 2:**
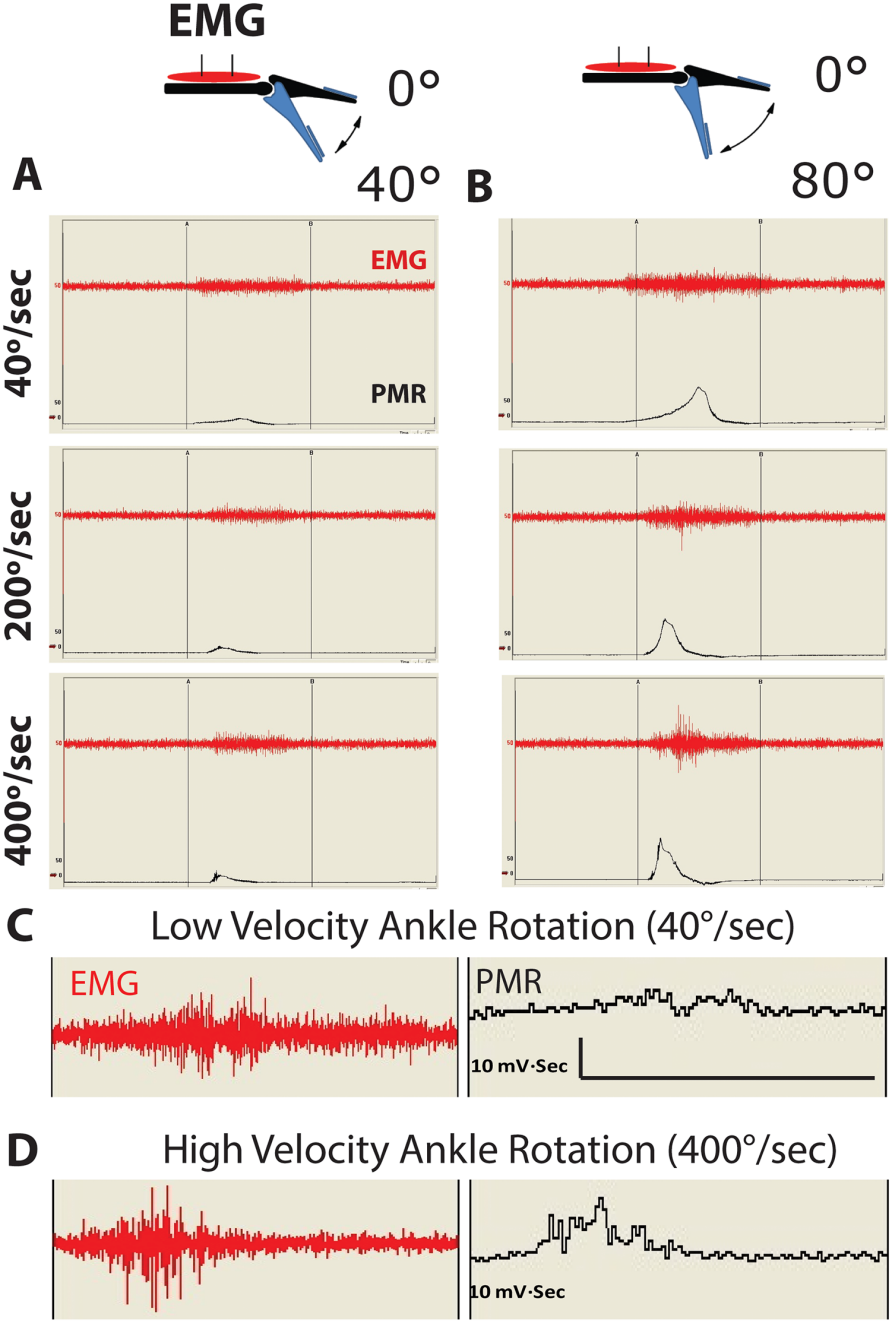
Potentiation of spinal hyper-reflexia by increased angle and velocity of ankle rotation. **(A, B, C, D)**—Comparing the effect of 40° to 80°of ankle rotation if ankle is rotated at 40, 200 or 400°/sec showed the most potent EMG response and corresponding increase in peripheral muscle resistance (PMR) at 80°of ankle rotation delivered at 400°/sec.

After spinal transection all animals were tested for the presence of spasticity in 15 days intervals. To identify spasticity, the ankle was rotated from a baseline position of 0° to 40° or 0° to 80° at a progressively increased velocity of ankle rotation (40, 200 and 400° /sec) and resulting changes in ankle resistance (PMR-peripheral muscle resistance) and gastrocnemius muscle activity (EMG) measured (Fig 2A and 2B). All testing was performed in fully awake animals. A consistent presence of spasticity were seen at intervals longer than 4 weeks after transection. This was expressed as ankle rotation-velocity-dependent increase in EMG and PMR and with most pronounced and consistent spastic response measured at a 400°/sec ankle rotational velocity with the ankle rotated from 0 to 80° (Fig 2A and 2B). In all subsequent experiments (i.e. the time course of muscle spasticity after spinal transection and all pharmacological studies) a 400°/sec of ankle rotational velocity with ankle rotated from 0 to 80° were used.

**Fig 3:**
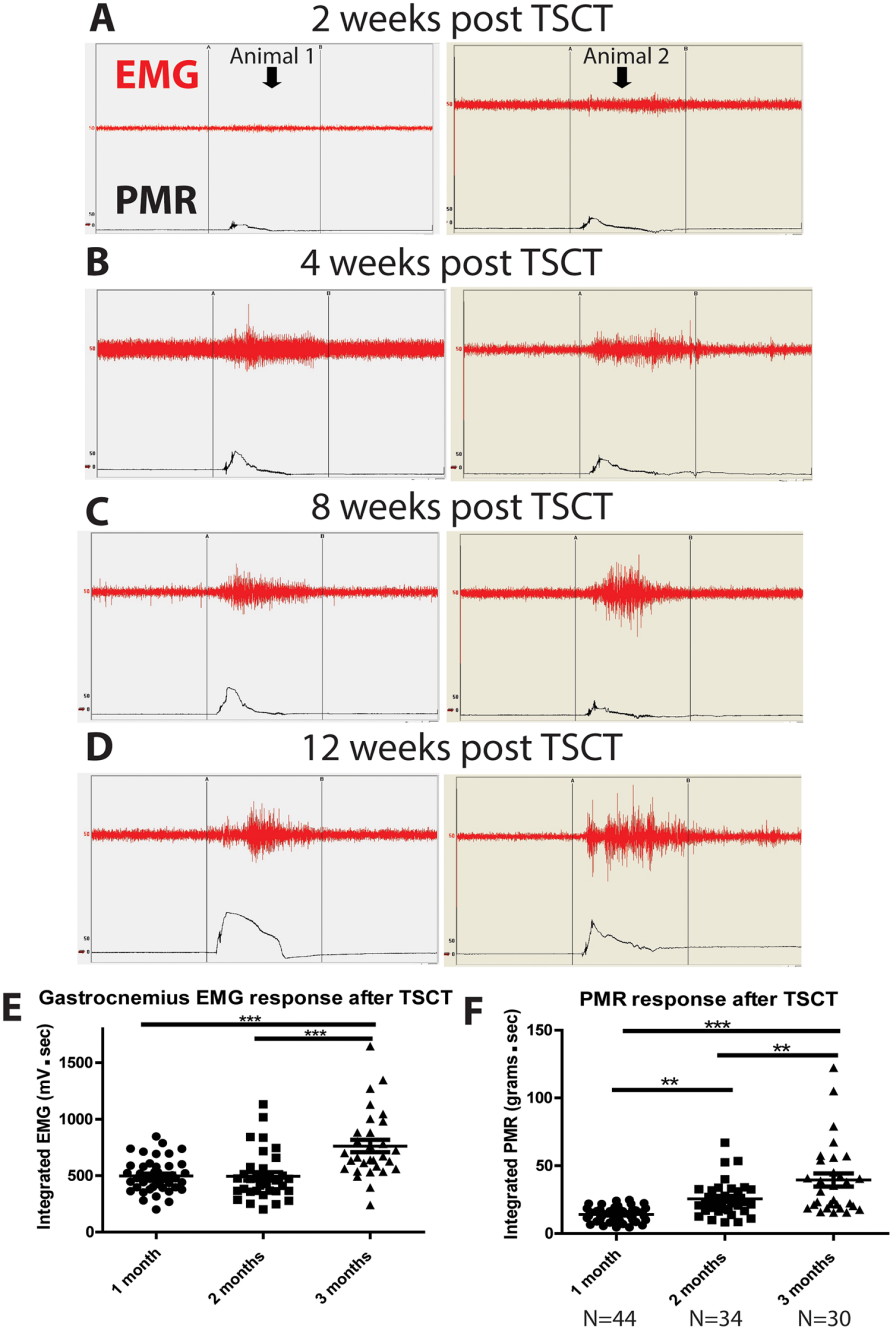
Post-spinal transection time-dependent increase in spinal hyper-reflexia. **(A, B, C, D)**—Measurement of EMG activity and PMR starting at 2 weeks after spinal transection showed a progressive increase in recorded responses with the most pronounced increase seen at 12 weeks after spinal transection. **(E, F)**—Statistical analysis showed a significant increase in both EMG response and PMR if compared across all 3 time points analyzed (i.e. 1, 2 and 3 months), (one-way ANOVA; Bonferroni post hoc; *** p< 0.001, ** p< 0.01).

Analysis of PMR and EMG response during ankle rotation showed a post-spinal transection time-dependent increase with the maximum response seen at 3 months post-transection (Fig 3A–3D).

Quantitative analysis of 5 sec integrated EMG response during ankle rotation showed similar EMG responses at 1 and 2 months after transection (496.9 ± 21.55 mV·s and 493.9 ± 37 mV·s; mean ± SEM) and this response were increased at 3 months (761.6 ± 54.69 mV·s; mean ± SEM), (Fig 3E). Analysis of PMR showed that at 1, 2 and 3 months post-transection there was a continually increased PMR from 13.72 ± 0.81 g·s, 24.58 ± 2.19 g·s and 38.73 ± 5.07 g·s (mean ± SEM), respectively (Fig 3F). Statistical analysis with one way ANOVA showed a significant time-dependent increases in both integrated EMG and PMR responses across the 3 month time course in which the rats were evaluated.

### Increase in H-reflex and the loss of rate-dependent-depression (RDD) in rats after TSCT

**Fig 4:**
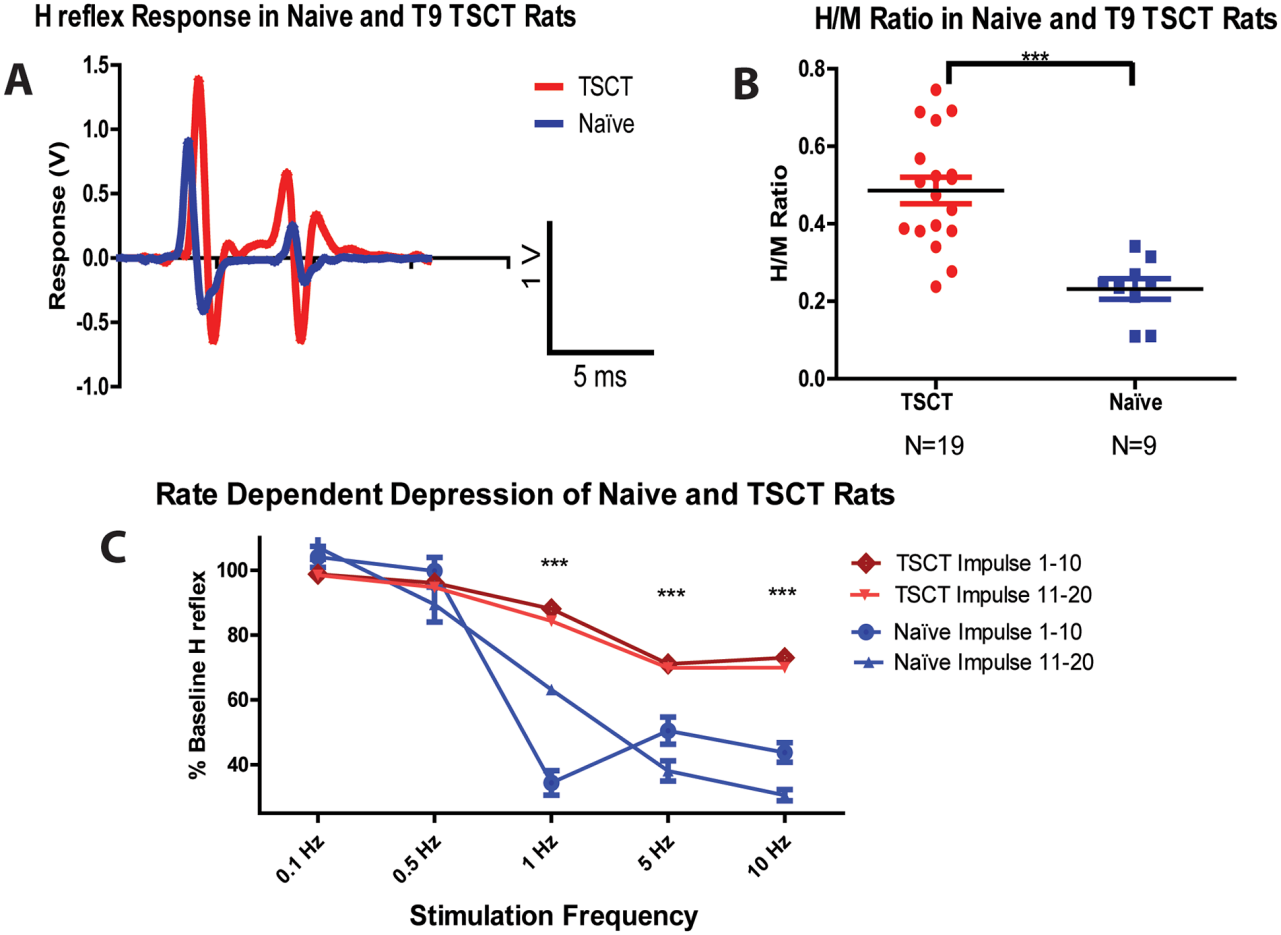
Increase in Hoffmann reflex and loss of rate-dependent depression (RDD) in spinally transected rats at 3 months after transection. **(A, B)**—Measurement of H-reflex and statistical analysis of the H/M ratio showed a significant increase in responses at 3 months after transection if compared to wild-type non-injured animals (unpaired two-tailed t-test; ***P< 0.001). **(C)**—Testing of RDD showed a significant loss of RDD in spinally-transected animals at stimulation frequencies of 1, 5 and 10 Hz (one-way ANOVA; Bonferroni post hoc; ***P< 0.001).

Changes in the H-reflex were analyzed at 3 months post-transection and compared to age-matched naïve controls. Statistical analysis showed a significant increase in the ratio of maximal H/M response in transected animals if compared to controls (0.23 ± 0.03 vs 0.50 ± 0.03, respectively), (Fig 4A and 4B; t-test, p<0.05). Analysis of rate-dependent depression (RDD) in naïve animals showed a progressive decrease in H-wave amplitude with increased rates of stimulation frequencies (1, 5, and 10 Hz). Statistical analysis of RDD at stimulation frequencies of 1, 5 and 10 Hz (10 repetitive stimuli) showed a significant loss of RDD in transected animals (control rats: 34 ± 3.8%, 50.49 ± 4.19%, and 43.76 ± 3.02% and T9 TSCT rats: 88.09 ± 1.27%, 71.09 ± 1.51%, and 72.99 ± 1.60% of baseline, respectively), (Fig 4C).

### Development of tactile stimulus-evoked spinal hyper-reflexia in spinally-transected animals

**Fig 5:**
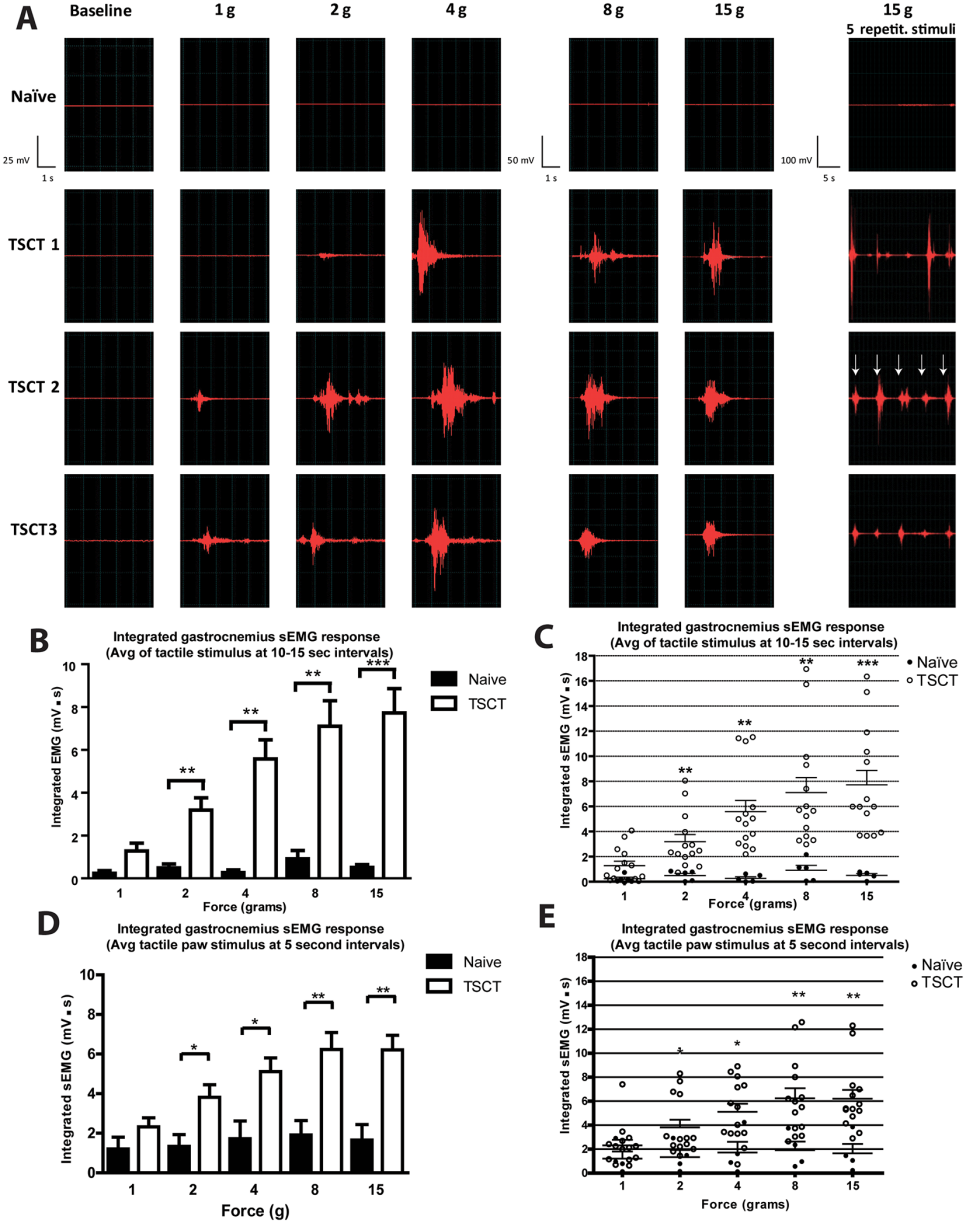
Development of tactile hypersensitivity in rats at chronic stages after spinal transection. **(A)**—Application of tactile stimuli (von Fray filaments; 1-15g) on the plantar surface of hind paw led to a clear EMG response measured by surface EMG electrodes from gastrocnemius muscle in animals at 3 months post-spinal-transection (TSCT 1, 2, 3). No response was seen in naive non-injured animals. Application of 5 repetitive stimuli at the same pressure (15g) and delivered every 5 seconds led to a consistent responses after each stimulus (A-right panels). **(B, C)**- Statistical analysis of repetitive (5x stimuli) tactile stimulus-evoked EMG responses separated by 10–15 seconds intervals showed a significant increase in transected animals (compared to naïve controls) at paw pressures between 2–15 grams (one-way ANOVA; Bonferroni post hoc; *-p< 0.05; **-p< 0.01; ***-p< 0.001). **(D, E)**- Statistical analysis of repetitive tactile stimulus-evoked (5x stimuli) EMG responses separated by 5 seconds intervals showed a comparable significant increase in animals with transection as seen after application of stimuli separated by 10–15 sec intervals (one-way ANOVA; Bonferroni post hoc; *-p< 0.05; **-p< 0.01; ***-p< 0.001).

To identify the presence of tactile stimulus-evoked hyper-reflexia, the EMG response was recorded from the gastrocnemius muscle using modified surface recording electrodes after the application of increasing pressures on the plantar surface of the right paw using von Frey filaments (1, 2, 4, 8 and 15 g). Test was performed in fully awake-restrained animals. In control naïve animals no EMG response was elicited with paw pressures up to 15 g (Fig 5A; naive). In contrast, in animals at 1–3 months after spinal transection a clear EMG response was recorded even after applying the lowest paw pressures tested (Fig 5A; 1–2 g; TSCT 1–3). This EMG response was further potentiated with incremental increase in applied paw pressures (compare Fig 5A; 1→ 15g). The supramaximal pressure stimulus was defined as a paw pressure which did not lead to a further increase in EMG response.

For quantitative analysis the average value of 5 integrated EMG responses recorded after applying the same paw pressure and separated by minimum of 10–15 second intervals between stimuli were used. At paw pressures of 2, 8, and 15 g control rats showed EMG responses of 0.71 ± 0.13 mV·s, 0.91 ± 0.38 mV·s, and 0.69 ± 0.13 mV·s while T9 TSCT rats had responses recorded at 3.18 ± 0.58 mV·s, 7.14 ± 1.19 mV·s, and 7.73 ± 1.14 mV·s (mean ± SEM), respectively (Fig 5B and 5C).

Statistical analysis with one-way ANOVA showed a significant increase in integrated EMG response after applying 2, 4, 8 and 15 g of paw pressure.

### Effect of repetitive tactile stimuli on EMG response in spinally transected animals

We next tested whether or not the application of 5 sequential tactile stimuli delivered at the same pressures and separated by 5 sec intervals will lead to a potentiation or suppression of EMG response. Representative recordings from one naïve and 3 transected animals demonstrating individual EMG responses after applying 5 repetitive paw pressure stimuli at supramaximal (15 g) intensity is shown in Fig 5A (right panels; 15 g—5 repeated stimuli). Statistical analysis of averaged 5 sec EMG signal recorded after applying 5 consecutive paw pressure stimuli every 5 seconds showed no significant increase in EMG response if compared to 5 consecutive stimuli applied in 10–15 seconds intervals (compare Fig 5B, 5C to 5D and 5E), (one-way ANOVA). A consistent EMG responses were seen across all 5 stimuli delivered at either 2, 8 or 15g of paw pressure; no potentiation or suppression of EMG response was measured.

### Effective anti-spastic effect after systemic treatment with clinically used anti-spastic agents

**Fig 6:**
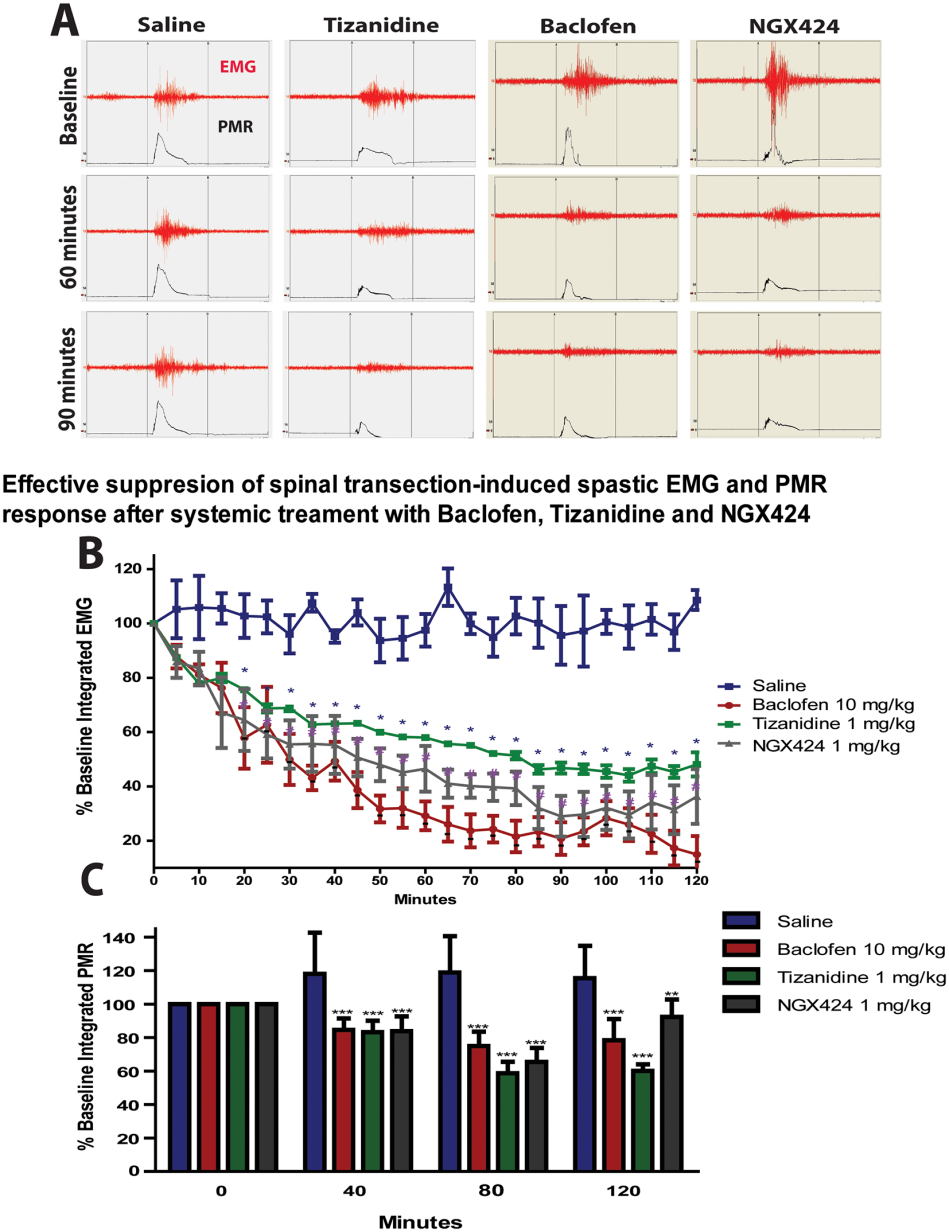
Effective suppression of ankle dorsiflexion-evoked EMG response and muscle resistance after systemic treatment with baclofen, tizanidine and NGX424. **(A)**- Representative recording of EMG response (EMG) and corresponding ankle resistance (PMR) in an animal injected systemically with saline, Tizanidine (1mg/kg), baclofen (10mg/kg) or NGX 424 (1mg/kg). **(B, C)**- Statistical analysis showed significant suppression of EMG response (B) and PMR (C) after treatment will all three drugs if compared to saline treated animals (repeated measures two-way ANOVA, Bonferroni post hoc; *-p< 0.05; **-p< 0.01; ***-p< 0.001).

To validate the clinical relevance of the rat Th9 transection model as a model of chronic spasticity and spinal hyper-reflexia we have used animals at 3 months after spinal transection and the effect of systemic (I.P.) baclofen (GABA_B_ receptor agonist; 10 mg/kg) and tizanidine (α_2_ adrenergic agonist; 1mg/kg) was tested. After drug administration there was a progressive decrease in measured EMG activity and muscle resistance (peripheral muscle resistance = PMR) during ankle rotation with the peak treatment effect for baclofen measured at 120 min (85.1% reduction) and at 105 min for tizanidine (55.9% reduction), (Fig 6A, 6B and 6C). Similarly as for baclofen and tizanidine, the systemic treatment with NGX 424 (AMPA/kainate antagonist, 10mg/kg) led to a progressive reduction of EMG response and muscle resistance with the peak effect seen at 90 min after drug administration (71.0% reduction). At 24 hours after treatment the EMG response returned back to baseline (not shown). In control-spastic saline-treated animals no change in EMG response or PMR was seen during the 2 hour post-injection recording.

**Fig 7:**
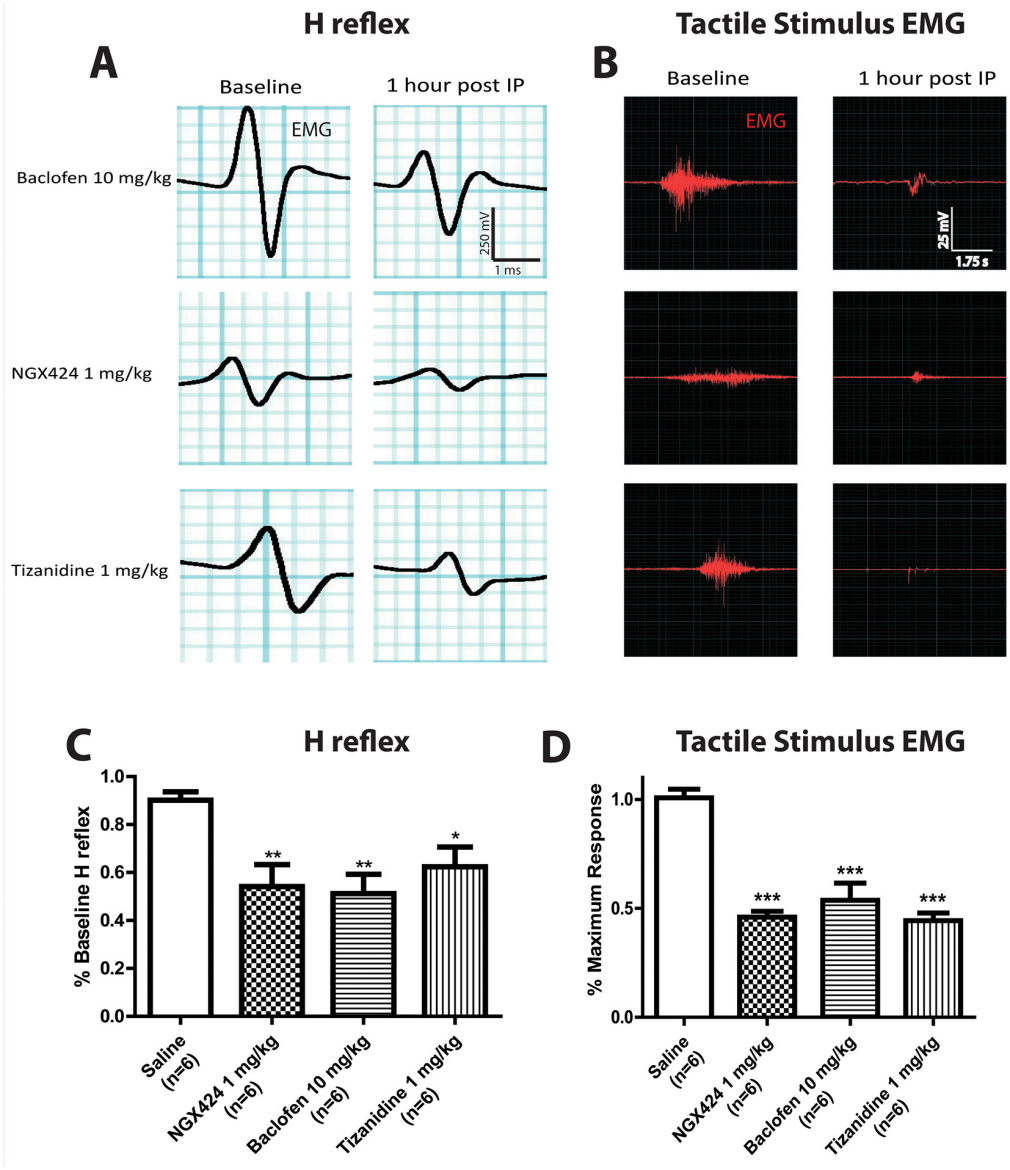
Effective suppression of Hoffmann reflex and spinal transection-induced tactile hypersensitivity after systemic treatment with baclofen, NGX 424 and tizanidine in rats with chronic spinal transection. **(A, B)**- Systemic treatment with baclofen (10mg/kg), NGX424 (1mg/kg) or tizanidine (1mg/kg) led to a clearly detectable suppression of H-reflex (A) and supramaximal tactile stimulus-evoked EMG response in rats at 3 months after spinal transection (B). **(C, D)**—Statistical analysis showed significant suppression of H-reflex (C) and tactile stimulus evoked EMG response if compared to saline-injected animals (one-way ANOVA; Bonferroni post hoc; *-p< 0.05; **-p< 0.01; ***-p< 0.001).

Analysis of pharmacological effects on H-reflex at 1 hr after using identical systemic treatment with baclofen, tizanidine, and NGX424 showed a significant suppression of H-reflex (Fig 7A–7C) with the average response being 51.2%, 62.8%, and 54.1% of baseline, respectively. Statistical analysis using one-way ANOVA showed a significant suppressive effect for all 3 drugs tested.

Similarly, testing the EMG response to a supramaximal tactile stimuli showed a suppression of EMG response at 1 hr after treatment (Fig 7B–7D) with the average gastrocnemius response being 53.7% (baclofen), 44.3% (tizanidine), and 46.0% (NGX424) of the baseline response. Statistical analysis using one-way ANOVA showed a significant suppressive effect for all 3 drugs tested.

### Immunofluorescent analysis of lumbar spinal cord sections at 3 months after spinal transection

**Fig 8:**
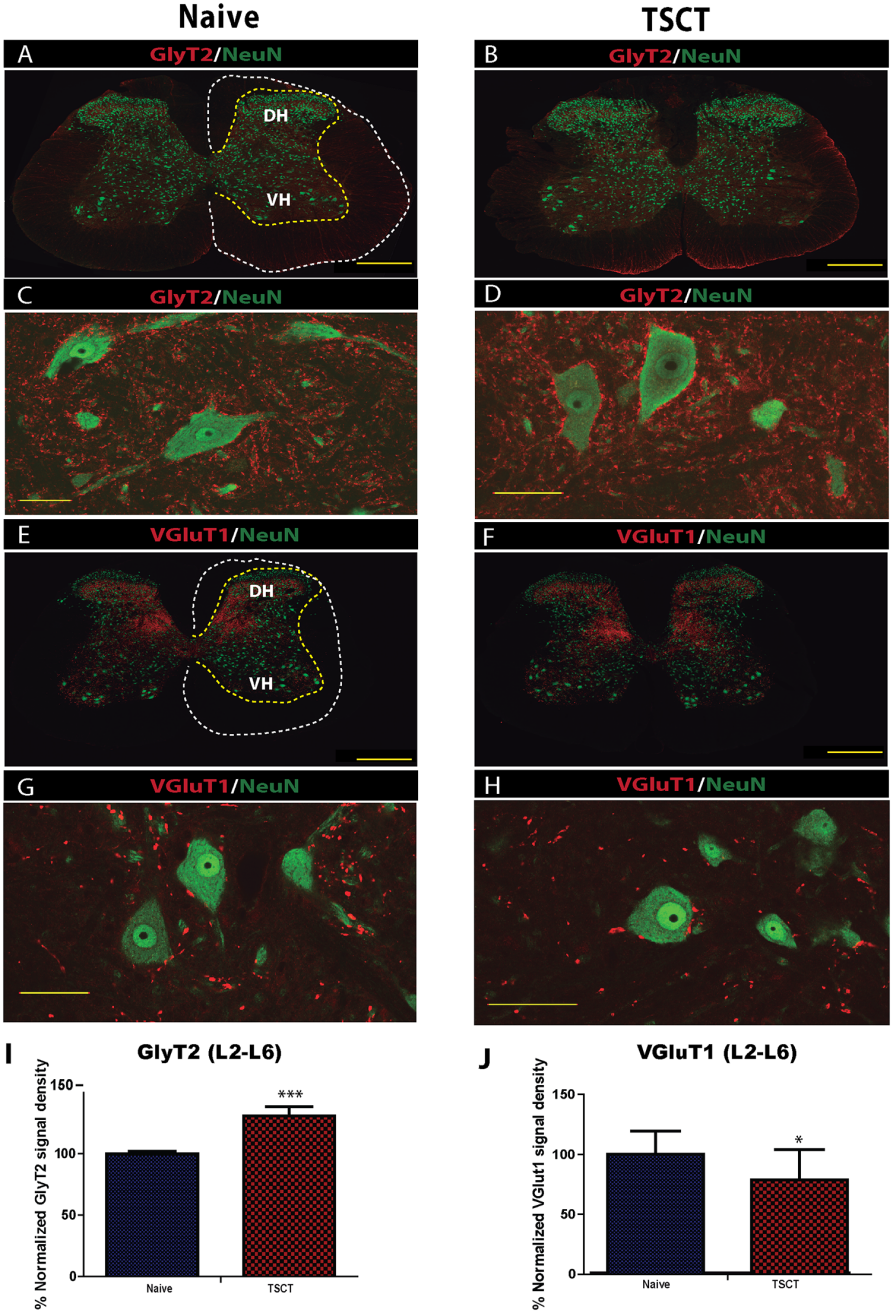
Quantitative and qualitative analysis of VGluT1 and GlyT2 expression in lumbar spinal cord (L2-L6) at 3 months post-spinal transection. **(A-H)**- Transverse spinal cord sections taken from L2-L6 segments in naïve and spinally transected animals (3 months post-transection) and double-stained with GlyT2/NeuN or VGluT1/NeuN antibodies. Normally appearing distribution of neurons and GlyT2/VGluT1 staining pattern can be seen in both control and spinally transected animals. **(I-J)**- Statistical analysis of GlyT2 density signal and VGluT1 puncta in the ventral horn showed a significant increase in GlyT2 expression and a significant decrease in VGluT1 puncta in chronically transected animals (t-test *-p< 0.05; **-p< 0.01; scale bar: A, B, E, F: 500 μm; C, D, G, H: 80 μm).

**Fig 9:**
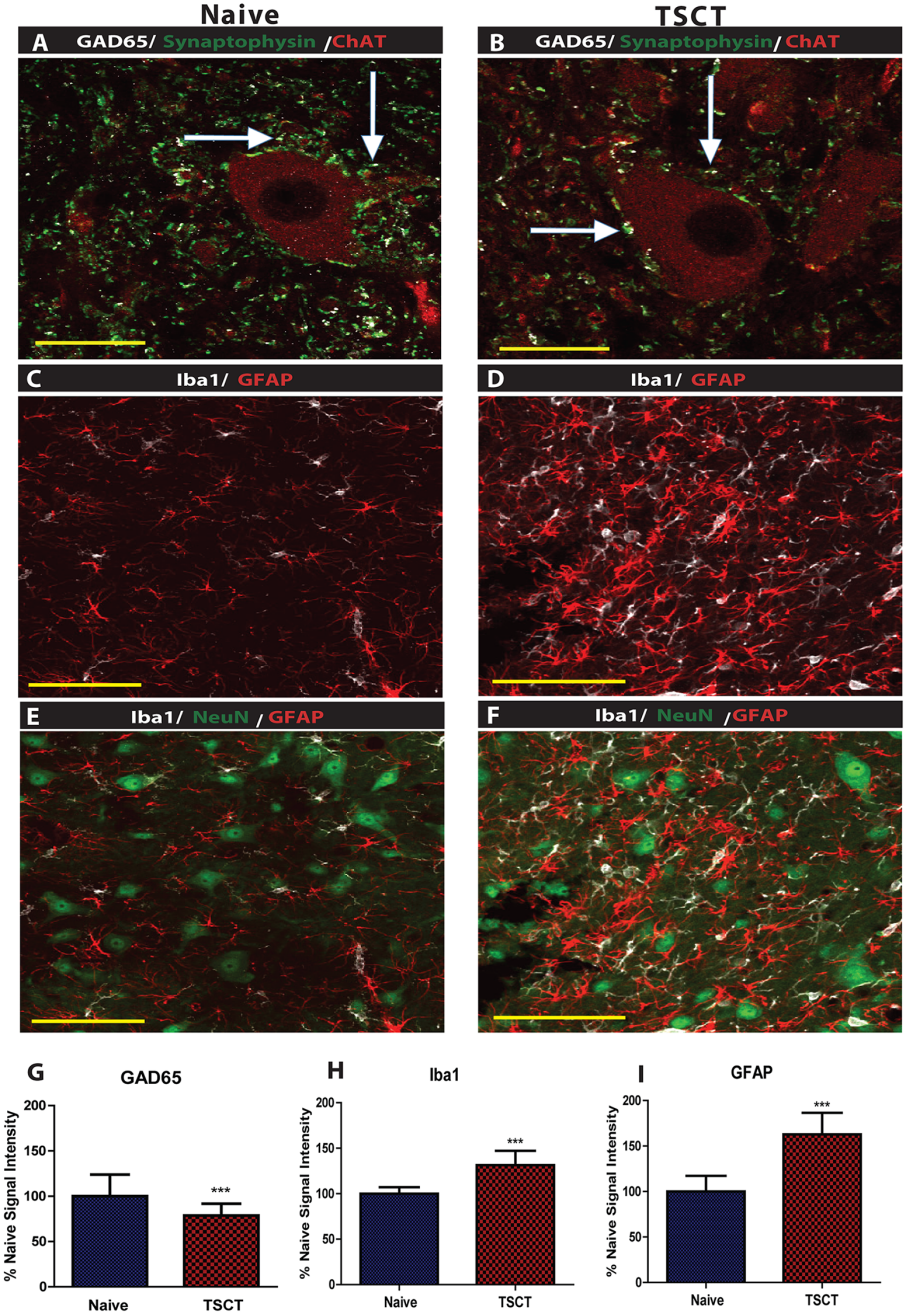
Immunofluorescence analysis of lumbar spinal cord sections at 3 months after spinal cord transection. **(A, B)**- Qualitative analysis of GAD65 and synaptophysin immuoreactivity showed normally appearing double-labeled synaptophysin/GAD65+ puncta (white arrows; confocal microscopy). **(C, D, E, F)**- Immunostaining with Iba1 and GFAP antibody (C, D) and NeuN (E, F) showed normally-appearing neurons, but an increase in IB1 and GFAP immunoreactivity in activated-hypertrophic astrocytes and microglial cells in lamina VII. **(G, H, I)**- Quantitative densitometric analysis of GAD65, GFAP and IB1 immunoreactivity showed a decrease in GAD65 and increase in GFAP and IB1 staining in TSCT animals if compared to naïve controls (t-test ***-p<0.001; scale bar: A, B: 50 μm C, D, E, F: 200 μm).

Transverse spinal cord sections taken from L2-L6 segments at 3 months after spinal transection were used and compared to control age-matched non-injured animals. Sections were stained with combinations of the following antibodies: VGluT1, GlyT2, ChAT, NeuN, GFAP, Iba1, GAD65/67 and synaptohysin. A systematic quantification of NeuN+ neurons and densitometry analysis of VGluT1 and GlyT2 was performed andcompared to naïve controls. Qualitatively the staining’s showed near normal immunofluorescent patterns for all antibodies used. The soma of large α-motoneurons, medium-sized interneurons and small interneurons stained with NeuN/CHAT antibodies showed normal appearance in all transected animals and was similar as seen in naive controls (Figs 8A, 8B, 9A and 9B).

Quantitative analysis of NeuN+ neurons in lamina VII in L2-L6 segments in T9 TSCT rats showed no significant difference when compared to naive controls (**Controls:** L2-96.5 ± 5.4, L3-119.3 ± 7.2, L4-111.3 ± 6.8, L5-137.8 ± 6.6, and L6- 143 ± 26.1; **Th9 TSCT:** L2-105 ± 4.5, L3-112 ± 7.5, L4- 124.3 ± 12.7, L5- 140.5 ± 9.6, L6- 177.5 ± 9.4). Quantitative analysis of GlyT2 immunodensity and VGluT1 puncta in the ventral horns of L4-L6 segments showed a significant increase in GlyT2 and decrease in VGluT1 expression in TSCT animals if compared to naïve controls (Fig 8I and 8J), (t-test). Quantitative densitometry analysis of GAD65/67-stained sections in the ventral horn showed a significant decrease in immunostained puncta in transected animals if compared to naïve, non-injured rats (Fig 9A, 9B and 9G), (t-test).

Quantitative densitometry analysis of GFAP/Iba1-stained sections showed a significant increase in the measured signal in the gray matter of spinally-transected animals (Fig 9D, 9F, 9H and 9I) compared to non-injured rats (Fig 9C, 9E, 9H and 9I), (t-test).

## Discussion

Over the past several decades numerous rodent models of chronic muscle spasticity have been developed and effectively used in either answering current questions about the pathophysiology of SCI-induced spasticity or screening new therapies to modulate muscle spasticity. In these models spinal hyper-reflexia is typically induced by a complete surgically-induced spinal transection, spinal contusion/compression injury or by lumbar spinal cord-targeted ischemic injury [21, 31]. In general in a majority of these studies relatively short-postinjury survival time (weeks) and qualitatively limited range of stimuli to trigger hyper-reflexive response were employed. Accordingly the clinical impact of these studies in providing a well characterized and extensively validated animal model of spasticity is rather limited. In our current study we employed a rat model of a complete Th9 spinal cord transection and characterized the time-dependent development of muscle spasticity evoked by computer-controlled ankle rotation. In addition the changes in Hoffmann reflex and the presence of peripheral tactile-stimulus-evoked spinal hyper-reflexia as assessed by peripheral EMG response was studied. The data from this study show a consistent presence of muscle spasticity and spinal hyper-reflexia at chronic (> 6 weeks) stages after spinal transection. This model appears to recapitulate several functional-diagnostic and pharmacological aspects of chronic muscle spasticity seen in human patients with spinal trauma-induced muscle spasticity including **i)** the appearance of velocity-dependent spasticity and tactile stimulus-evoked hyper-reflexia and **ii)** effective suppression of spasticity and hyper-reflexia by a clinically used and extensively validated anti-spastic agents.

### Appearance of time-dependent muscle spasticity and hyper-reflexia after spinal transection

In our current study 2 principal tests were used to identify the presence of spinal hyper-reflexia in animals after spinal transection. First, ankle rotation-evoked changes in EMG activity and corresponding ankle resistance were measured after applying a progressively increased ankle-rotation velocities in fully awake animals. Using this test an ankle-rotation-velocity-dependent increase in EMG response and ankle resistance was seen. The spasticity response evoked by ankle rotation showed post-transection time-dependent increase with the maximum response recorded at 3 months post-transection. Second, the effect of tactile stimuli applied on the surface of the paw was tested in its potency to trigger spinal-reflex-mediated EMG response. As we have reported in our previous study [38] a clearly developed tactile hypersensitivity, expressed as EMG response after applying low tactile paw pressures (as low as 1 g) was measured. This tactile-stimulus-evoked hypersensitivity response was consistently recorded as soon as 4 weeks after transection and persisted for the duration of study (i.e. 3 months post-transection). A progressive increase in applied paw pressure led to increased EMG response from the gastrocnemius with the maximum response typically elicited with about 8–15 g of paw pressure. In a separate group of animals we have measured the continuing and quantitatively unchanged presence of spasticity and hyper-reflexia for up to 14 months post spinal transection (unpublished observation).

These observations are similar as described in other rodent models of spinal injury-evoked spasticity. First, using a complete S2 transection model in rat a progressive appearance of muscle stretch and cutaneous stimulus-evoked muscle spasm were seen at intervals longer than 2 weeks after spinal transection [21]. In the same study, an electrical stimulation or light hair or skin touch was used to activate a low threshold cutaneous afferents. It was also demonstrated that a windup of spinal reflexes developed in chronically transected animals after repetitive low-threshold peripheral stimulation and is associated with reversal of cutaneous reflexes from inhibitory to excitatory. The precise mechanism leading to the development of these pathological responses is not clearly understood but it is believed that it may include the loss of tonic descending inhibition and resulting decrease in response threshold of spinal first-order neurons to peripherally-applied stimuli [39]. In addition the emergence of both sodium and calcium-specific persistent inward currents on motor neurons leading to stronger and more prolonged depolarization potentials was measured caudal to the injury site [40].

In our study, an increase in the H reflex (as well as H/M ratio) and significant loss of rate-dependent depression (RDD) was measured at 3 months post-spinal transection when compared to control rats. Similar increases in H-reflex measured from acute to chronic stage after spinal sacral transection have been reported [21]. Other studies in rats and humans which studied reflex responses have demonstrated alterations in the H reflex response, as well as a decrease in the rate-dependent depression following high frequency stimulation similar to what we have seen in our current study [41, 42]. To identify a potential contribution of segmental interneuronal loss in the observed increase in H-reflex and loss of presynaptic inhibition we quantified the number of NeuN- stained interneurons in lamina VII in animals at 3 months post-spinal transection (i.e. in the time frame which was characterized by a peak spastic muscle response, H-reflex and loss of RDD). No significant loss of interneurons was seen. Based on these data we speculate that the loss of descending facilitatory-inhibitory tone likely represents the primary mechanism leading to the spasticity and hyper-reflexia in this model. In contrast a decrease in number of GABA-ergic neurons in T11 contusion injury model in mice was reported. In the same model the development of mechanical hyperalgesia (similar as seen on our current study) and thermal hyperalgesia below the level of the lesion was also seen [43].

### Contribution of Ia and Aβ afferents in triggering EMG response after peripheral stimulation in chronic spinally-transected animals

As outlined, two different stimulation modalities were used in our current study to identify the presence of muscle spasticity and spinal hyper-reflexia. In the **first** test a computer-controlled ankle rotational system was used. Here, the ankle is rotated using a firmly placed “paw attachment plate” which make continuous contact with the plantar surface of the paw. After the rotation is initiated a pressure is exerted on the paw surface until the rotation has completed. The EMG response is recorded before, during and after paw rotation. Using this test we have demonstrated the presence of clear ankle-rotation-evoked EMG response presumably mediated in part by the activation of stretch reflex (i.e. Ia afferent activation during muscle stretch). In the **second** test the effect of progressively increased paw pressures applied with von Frey filaments was tested in its potency to trigger gastrocnemius muscle EMG response. As shown even low paw pressures (1–2 g) were effective in triggering spinally-mediated EMG responses suggesting the presence of high degree of tactile Aβ afferents-mediated hypersensitivity. However, because of the demonstrated extremely developed tactile hypersensitivity in this model it is likely that the EMG response recorded during the ankle rotation represents a mixed Aβ and Ia input-dependent response. The Aβ afferents are likely activated first during the initial phase of ankle rotation before a full stretch-evoked Ia afferent activation is achieved. To address this hypothesis and to delineate the contribution of Aβ and Ia afferents in recorded response during ankle rotation we have blocked the paw tactile component by plantar nerve block (2% lidocaine) before ankle rotation. After the nerve block the recorded EMG response was substantially reduced during the initial phase of ankle rotation (i.e. likely mediated by cutaneous Aβ afferents and other high-threshold afferents) and was then followed by a delayed EMG response clearly recorded at the peak of muscle stretch (S1 Fig). These data suggest that the exacerbated EMG response recorded in chronic spinalized animals during ankle rotation in fact represents a mixed Aβ/Ia afferents activity-mediated response, which should be interpreted as Ia/Aβ-mediated spinal hyper-reflexia and spasticity. It is important to note that both of these qualitatively distinct afferent input-triggered functional tests have been employed previously in independent studies and have demonstrated an increase in the EMG and torque responses during ankle rotation, and increase in tactile stimulus-evoked responses in a spinalized rats [25, 44].

### Pharmacology effects on the muscle spasticity state after spinal trauma

To validate the clinical relevance of current spinal transection model of muscle spasticity the effect of systemically-delivered baclofen (GABA_B_ receptor agonist) and tizanidine (α_2_ adrenergic agonist) (i.e. clinically well-established anti-spasticity agents) were tested. After treatment with both compounds a clear anti-spasticity effect and suppression of spinal hyper-reflexia was seen. Using rat spinal ischemia model of spasticity we have previously reported on a comparable antispasticity effect after systemic or intrathecal treatment with baclofen and tizanidine [29, 31, 32]. These data are consistent with numerous clinical studies which show a potent anti-spasticity effect after systemic or spinal (intrathecal) treatment with baclofen and tizanidine in patients with spasticity of different etiology including multiple sclerosis, stroke, spinal cord injury, and syringomyelia [45–49].

In addition to tizanidine and baclofen treatment the effect of systemic treatment with NGX424 (a novel AMPA receptor antagonist) was tested in animals at chronic stages of spinal transection-induced spasticity. A potent but transient anti-spasticity effect was seen after NGX 424 treatment. The potency of NGX424 anti-spasticity effect seen in animals after spinal transection was similar as we have previously reported in spinal ischemic model of spasticity as well as in spinal ischemic-spastic animals with fully developed baclofen tolerance [50, 51]. These data demonstrate that similarly as seen in spinal ischemic spasticity the AMPA receptor complex play an important role in the evolution and maintenance of spasticity at chronic stages after spinal transection. Accordingly the use of treatment strategies aimed at decreasing segmental AMPA receptor activity (such as AMPA receptor silencing or antagonism) can have a potential in providing anti-spastic effect in patients with muscle spasticity. Jointly these data indicate that a rat chronic spinal transection model of muscle spasticity show a potent anti-spasticity response to a clinically well-established anti-spasticity treatment and represent an appropriate animal model for testing a new anti-spasticity compounds to be used in perspective human clinical trials.

### Pros and cons in using a rat complete Th9 spinal transection model as a model of chronic spasticity

The major limitation of the current model appears to be a relatively labor intensive animal care which is required during the first 2–3 weeks post-transection. Based on our animal care experience with over 130 transected animals which survived over 3 months after spinal transection the following animal care principles were established and vigorously followed: i) bladder expression every 12 hours, ii) the addition of antibiotics (Cefazolin (s.c.), SMZ (drinking water) or penicillin/streptomyocin (s.c.)) during the first 14 days post OP and once the urine is seen as either cloudy or bloody, iii) the administration of either lactated ringers or 0.9% saline subcutaneously in dehydrated rats. Following of these procedures in our post-operative animal care aid in the prevention of urinary tract infections, urethral obstruction and formation of bladder calculi. While animals were kept in cages with sani-chip soft bedding (changed twice a week) the development of lower extremities ulcers was seen in 10% of animals. Identified ulcers were effectively treated using 0.05% chlorohexidine washes and topical antibiotic (bacitracin, neomycin, and polymyxin B). From the perspective of the animal care outlined above, the use of sacral (S2) transection model is clearly superior by eliminating the post-spinal transection-induced bladder paralysis when only tail musculature is hyper-reflexive [21]. The advantage of using a complete Th9 transection model appears to be its ability to recapitulate a multisegmental hyper-reflexia extending from lower thoracic to lumbo-sacral segments and its complexity in affecting equally large extensor and flexor muscle groups of the hindlimbs, i.e. characteristics seen in human patients with spinal trauma induced spasticity [2, 52, 53]. In addition from the perspective of development of new therapies based on spinal parenchymal or lumbar intrathecal cell or gene delivery the use of Th9 model appears to be advantageous in terms of providing more easily accessible intrathecal space and larger spinal parenchyma to perform segment-targeted injections.

### Histopathological changes in lumbar spinal cord after transection and postulated mechanism of resulting muscle spasticity

To address the potential role of the altered balance between inhibitory vs. excitatory spinal segmental activity we have systematically analyzed and quantified the expression in VGluT1, GAD65/67, and GlyT2 in the lumbar spinal cord sections in both the control and spinally-transected spastic animals. The VGluT1 (vesicular glutamate transporter 1) has been shown to be expressed primarily in low-threshold cutaneous and proprioceptive myelinated (Ia) afferents [54]. The primary function of GlyT2 (glycine transporter 2) is its role in vesicular glycine packaging in the presynaptic terminals of glycine-ergic neurons [55, 56]. Histopathological analysis of T9 TSCT lumbar spinal cords at 3 months post-transection showed a statistically significant decrease in VGluT1 and GAD65/67, while a significant increase could be seen in GlyT2 immunostaining. While the functional relevance of these changes in terms of its contribution to potentiation or suppression of spinal hyper-reflexia is not defined, we hypothesize that the changes in the activity of these systems can potentially have the following implications. First, a decrease in VGluT1 expression may represent a counterbalanced compensatory response to prevent excessive excitatory overdrive following a downregulation GAD65/67-mediated GABA-ergic inhibitory tone. Second, an increase in GlyT2 expression can similarly be the result of the disinhibited system driving toward facilitated/increased glycinergic inhibition to counterbalance increased excitatory drive. The loss of GlyT2 activity in knockout animals is associated with spasticity, hypertonia, and rigidity [55, 56]. In addition in our recent study using the same spinal transection model we have found that a long-term spinal GlyT2 silencing leads to an exacerbated spinal hyper-reflexia suggesting that the GlyT2 system still play an active inhibitory role in chronically spinalized rats [57]. Based on these data we speculate that the increase in GlyT2 expression and activity can lead to an increased glycinergic inhibition of ventral α-motoneurons. The fact that these animals still show a prevalence of excitatory drive also indicate a continuing excitatory-inhibitory imbalance. The down-regulation of VGluT1 and upregulation of GlyT2 could be the combined adaptive response that leads to a decrease in the excitatory effect on motor neurons. In addition, several other mechanisms have been postulated to play a role in increased α-motoneuronal excitability after spinal trauma. It has been demonstrated, for example, that the potassium-chloride cotransporter KCC2 is downregulated in α-motoneuron membranes in spinally-injured rats. This leads to depolarization of the Cl- equilibrium potential and a reduction in strength of gamma-aminobutyric acid(A) (GABA A) and glycine receptor-mediated postsynaptic inhibition [2, 52, 53].

We have also demonstrated a significant increase in GFAP and IB1 immunoreactivity in animals with spinal transection-induced hyper-reflexia. Previous studies have suggested that this is a result of the injury-induced proinflammatory cytokine release such as IL-1 beta, IL-6, and TNF alpha, which leads to activation and proliferation of microglia and astrocytes [9–12]. In turn, the activation of glial cells has been shown to be associated with the secondary release of several neurohormones/neuromodulators which may potentiate neuronal excitability. These may include glutamate (excitatory amino acids), D-serine, and ATP, all of which can contribute to the activation and potentiation of excitatory neuronal drive and the downstream calcium-dependent and -independent protein kinases[13–18]. These changes are believed to contribute to hypersensitized spinal responses to otherwise physiological peripherally-applied stimuli [19, 20] and are clinically presented as chronic pain and/or general spinally-mediated hyperexcitability including muscle spasticity and rigidity [19, 58, 59].

## Conclusions

We characterized a chronic rat spinal transection-induced model of muscle spasticity and spinal hyper-reflexia. The presence of spasticity and hyper-reflexia was consistently measured at intervals longer than 3 weeks after spinal transection and were characterized by the presence of i) velocity-dependent muscle spasticity, ii) profound tactile-stimulus evoked hyper-reflexia and iii) increase in Hoffmann reflex. These data demonstrate that a chronic Th9 spinal transection model in adult rats represents a reliable experimental platform to be used in studying the pathophysiology of chronic spinal injury-induced spasticity. In addition a consistent anti-spastic effect measured after treatment with clinically effective anti-spastic agents indicate that this model can effectively be used in screening new anti-spasticity compounds or procedures aimed at modulating chronic spinal trauma-associated muscle spasticity.

## Supporting Information

S1 Fig Effect of plantar nerve block on tactile stimulus-evoked and ankle rotation-evoked EMG activity and ankle resistance. **(A, B)**- a consistent PMR and EMG response during ankle rotation and after application of paw tactile stimulus (15 g) can be seen in fully awake animals at 3 months post spinal transection. **(C, D)**- after plantar nerve block the EMG response during ankle rotation is reduced and the peak response is seen at the end of rotation i.e. at the peak of muscle stretch. The paw tactile stimulus-evoked response is completely lost after nerve block. **(E, F)**- After induction of isoflurane anesthesia both EMG responses evoked by ankle rotation or paw tactile stimulus are lost.
(TIF)

S2 Fig Experimental setup for recording of paw tactile stimuli-evoked EMG response in gastrocnemius muscle in fully awake-restrained rat. **(A, B)**—Animals are placed into a PVC tube (6 cm in diameter; 30 cm length) and their right paw taped to the surface of the table. **(C)**- To evoke a tactile stimulus-evoked EMG response a calibrated force is applied on the plantar surface of the extended paw (blue arrow) using von Fray filaments and EMG response recorded from gastrocnemius muscle using two surface EMG electrodes (3 mm wide and 2 cm long; red arrows).
(TIF)

## Abbreviations

ANOVA: Analysis of variance
ChAT: Choline acetyltransferase
DAPI: 4',6-diamidino-2-phenylindole
DPS: Degrees per seconds
EMG: Electromyography
G: Gauge
GAD65: glutamic acid decarboxylase isoform 65
GFAP: Glial fibrillary acidic protein
GlyT2: Glycine transporter 2
Hmax: Maximum H wave
H-reflex: Hoffmann reflex
Iba1: Ionized calcium-binding adapter molecule 1
IM: Intramuscular
IP: Intraperitoneal
L: Lumbar segment
mA: milliAmperes
Mmax: Maximum M wave
NeuN: general neuronal nuclei marker
PBS: Phosphate buffered saline
PMR: Peripheral muscle resistance
PO: Per os
RDD: Rate-dependent depression
S2: Sacral segment 2
SC: Subcutaneuous
SCI: Spinal cord injury
SD: Sprague-Dawley
SMZ: Sulfamethazole
Th9: Thoracic segment 9
Th13: Thoracic segment13
Th8: Thoracic segment 8
TSCT: Transected
VGluT1: Vesicular glutamate transporter 1
VGluT2: Vesicular glutamate transporter 2
