# Supplementary figures and images for: Thoracic 9 Spinal Transection-Induced Model of Muscle Spasticity in the Rat: A Systematic Electrophysiological and Histopathological Characterization
Source: PLoS One. 2015 Dec 29;10(12):e0144642. doi: 10.1371/journal.pone.0144642 (PMC4705098; doi:10.1371/journal.pone.0144642)

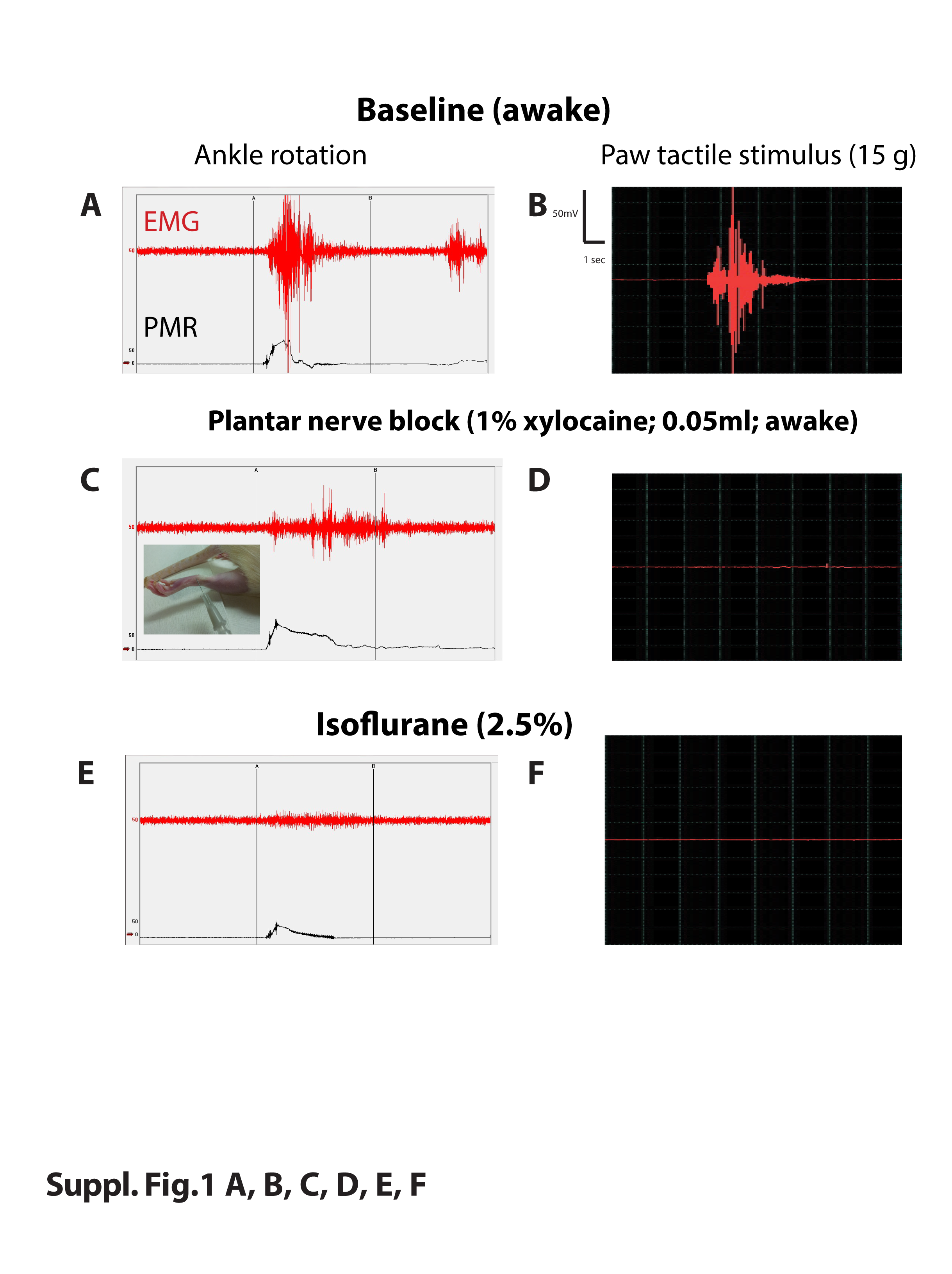

Supplement: S1 Fig — (A, B)- a consistent PMR and EMG response during ankle rotation and after application of paw tactile stimulus (15 g) can be seen in fully awake animals at 3 months post spinal transection. (C, D)- after plantar nerve block the EMG response during ankle rotation is reduced and the peak response is seen at the end of rotation i.e. at the peak of muscle stretch. The paw tactile stimulus-evoked response is completely lost after nerve block. (E, F)- After induction of isoflurane anesthesia both EMG responses evoked by ankle rotation or paw tactile stimulus are lost. (TIF) [file pone.0144642.s001.tif]

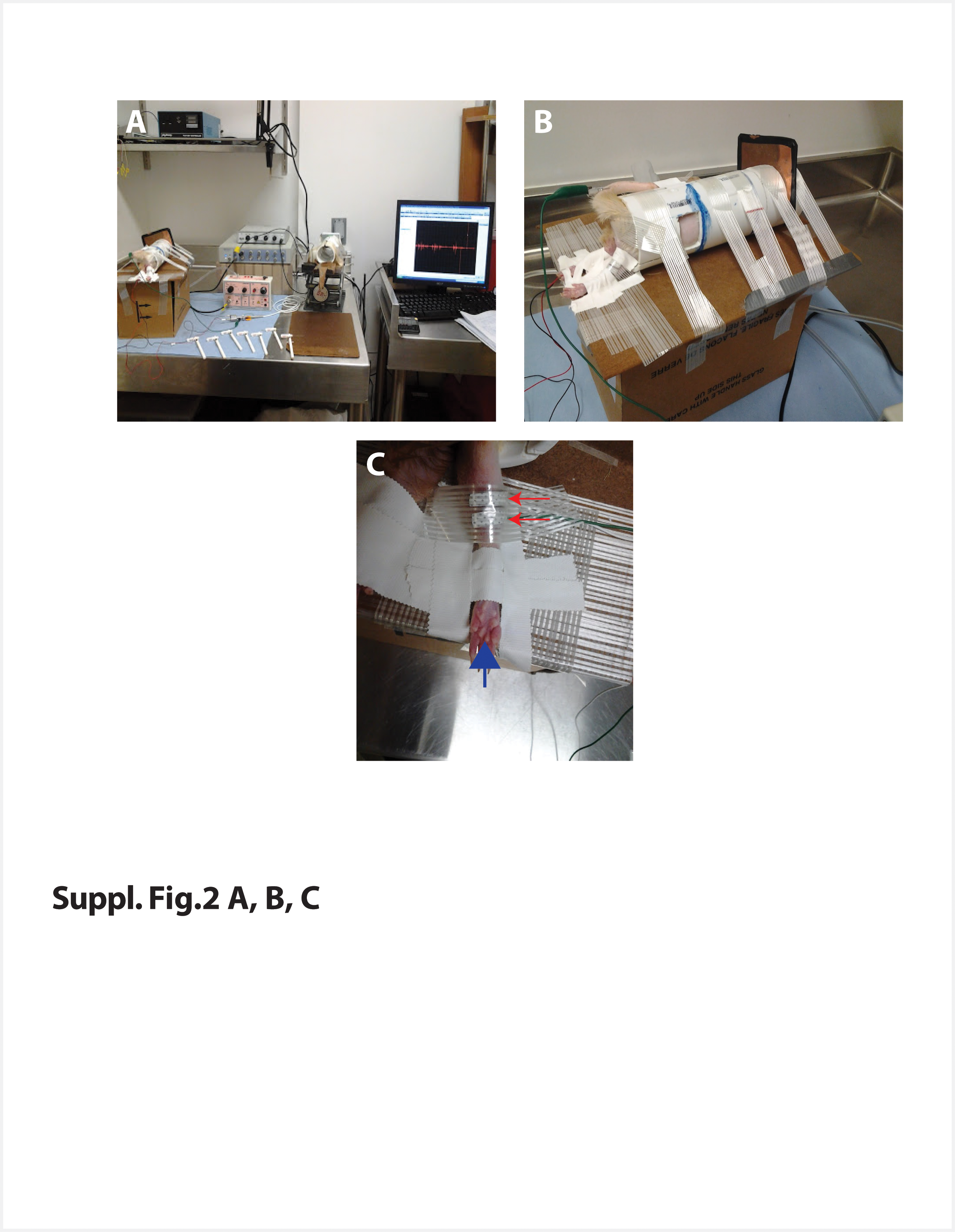

Supplement: S2 Fig — (A, B)—Animals are placed into a PVC tube (6 cm in diameter; 30 cm length) and their right paw taped to the surface of the table. (C)- To evoke a tactile stimulus-evoked EMG response a calibrated force is applied on the plantar surface of the extended paw (blue arrow) using von Fray filaments and EMG response recorded from gastrocnemius muscle using two surface EMG electrodes (3 mm wide and 2 cm long; red arrows). (TIF) [file pone.0144642.s002.tif]
